# The Impact of Family Income on Body Mass Index and Self-Rated Health of Illiterate and Non-illiterate Rural Elderly in China: Evidence From a Fixed Effect Approach

**DOI:** 10.3389/fpubh.2021.722629

**Published:** 2021-09-17

**Authors:** Yu Xin, Xiaohui Ren

**Affiliations:** Department of Health Behavioral and Social Medicine, West China School of Public Health and West China Fourth Hospital, Sichuan University, Chengdu, China

**Keywords:** rural elderly, family income, body mass index, self-rated health, fixed effects

## Abstract

**Background:** Rural communities worldwide are experiencing the most significant levels of aging. Most rural elderly have no stable pension, and leading family income plays an indispensable role in the life security of rural elderly. This study aims to investigate whether the association between annual family income per capita and body mass index (BMI) and self-rated health (SRH) in rural elderly is moderated by education during fast economic development.

**Methods:** We chose the fixed-effects model to analyze the impact of the annual family income per capita change on BMI and SRH based on a large, nationally representative longitudinal dataset of rural respondents aged above 60 of the China Family Panel Studies (CFPS) from 2010 to 2018.

**Results:** Six hundred and fifty-eight were eligible for inclusion in our analysis in CFPS. The median age of participants was 65 years in 2010, and 379 (57.60%) participants were male. Self-rated health increased with higher the logarithmized family income per capita among the rural illiterate elderly (β = 0.0770; 95% CI = 0.0065–0.1473). Body mass index increased with higher the logarithmized family income per capita among the rural elderly (β = 0.1614, 95% CI: 0.0325–0.2903), and it was more evident among the illiterate elderly (β = 0.2462, 95% CI: 0.05519–0.4372).

**Conclusion:** Family income has an impact on BMI and SRH moderated by education level among rural elderly in China. These results contribute to developing more targeted strategies in the context of a developing country. In addition, it also reminds us to consider the differences in the educational level of the elderly in rural areas when examining the relationship between family income and health.

## Introduction

With the decline of fertility and mortality, the number of elders continues to increase rapidly. It is estimated that between 2017 and 2050, the proportion of the world's population aged 60 and above will nearly double from 926 million to 2.1 billion (1). The problem of aging is particularly evident in Asia. From 2015 to 2050, the total number of people aged 80 and above in 23 Asian countries is expected to quadruple (2).

Rural communities worldwide are experiencing the most significant levels of aging in place, including many developed countries and developing countries, such as China, India, Japan, Indonesia, Australia, the United States (3–7). Compared with the urban elderly, the rural elderly have limited economic and material resources (8, 9). Under the background of economic and social transformation and rural structural adjustment, the rural economy of many developing countries has developed rapidly (10–13). Some developing countries, such as Brazil, South Africa, Mexico, and China, have launched government transfer or social pension programs for elderly who have not previously covered pensions (14). Nevertheless, the elderly in rural areas, especially in less developed regions, is expected to receive less pension than those in urban counterparts (15). Therefore, family income has played an indispensable role in life support for the rural elderly.

Previous studies confirmed a relationship between family income and health (16, 17). The reason may be that income affects health through quality housing, social networks and relationships, health-related knowledge, diet choices, and sports activities in safe communities (18). Most previous studies of the association between family income and health have used regression modeling applied to cross-sectional data (19–22). We cannot be confident that the associations uncovered in these studies are not spurious by this methodological approach. In addition to methods, the choice of outcome indicators will also affect the results. Veenstra and Vanzella-Yang found that the change of family average income impacted health (18), but it only takes self-rated health (SRH) as an outcome indicator and lacks the objective outcome indicator to evaluate health.

The outcome indicators used in this study were body mass index (BMI) and SRH. Body mass index was regarded as an objective indicator of health outcomes. Moreover, the health burden of obesity has been widely recognized, and it is the leading risk factor for many adverse health consequences, such as cardiovascular disease, diabetes, and depression. Therefore, it has been the focus of many researchers in social science and public health to clarify the reasons for weight gain (23, 24). Self-rated health was used as an outcome indicator to evaluate the subjective health of the elderly. It is also a concise and straightforward public health indicator to evaluate the overall health status in epidemiological studies (20), and it is also can be used as a predictor of subsequent mortality and health issues (25, 26). This study aims to find the relationship between the growth of family income and the health changes of the elderly in rural areas based on a large, nationally representative longitudinal dataset of rural respondents aged above 60 from three waves of the China Family Panel Studies (CFPS) from 2010 to 2018. Because the education level of the elderly did not change, this study hopes to focus on whether annual family income per capita has different effects on BMI and SRH of the elderly with different educational levels.

## Methods

### Sample and Data Collection

The data were derived from the CFPS. China Family Panel Studies is a biennial longitudinal survey conducted by the Institution of Social Science Survey at Peking University. This investigation launched in 2010 with five waves of publicly released datasets. The samples covered 25 provinces, accounting for 95% of the total population of China. The contents of CFPS are rather typical, covering the demographics, socioeconomic condition, education, and health of respondents.

The Biomedical Ethics Review Committee of Peking University approved CFPS, and all participants were required to provide written informed consent. The ethical approval number was IRB00001052-14010.

Since the fixed-effect regression studied the relationship between the change in dependent variables and those independent variables across each wave, we selected 658 participants according to the following criteria: (1) aged 60 and above in 2010, (2) rural elderly (respondents were categorized into urban and rural residents according to their household living regions defined by National Bureau of Statistics of the People's Republic of China), and (3) had not missing values of dependent or independent variables in all three waves (2010, 2014, and 2018).

### Variables

#### Dependent Variable

The health outcomes selected in this paper are SRH and BMI, considered from subjective and objective aspects.

The dependent variable was SRH. Respondents were asked, “in general, would you say your health is excellent, very good, good, fair, or poor?” We coded this variable as 5 = excellent, 4 = very good, 3 = good, 2 = fair, and 1 = poor.

Body mass index, a continuous measure of body weight relative to height, was calculated as body weight in kilograms divided by squared height in meters (kg/m^2^).

#### Independent Variable

Family income was measured by annual family income per capita (taking the natural log and deflating based on the 2010 CPI). Family income of CFPS included wage income, business income, transfer income, property income, and other income. Then, we divided the family income by the family size to get the family per capita income.

#### Control Variable

Socio-demographics included sex, age, marital status, and the number of years of education. Age and the number of years of schooling were continuous variables. Marital status and chronic diseases weredefined as binary variables. Marital status was divided into married and divorced/widowed/unmarried, and chronic disease was divided into with and without.

Illiterate refers to the older adults who have not received a formal education, that is, the number of years of education was 0. Non-illiterate refers to the older adults who have received a formal education, that is, the number of years of education was more than 0.

### Analysis Strategy

Fixed effect model and random effect model can explain the estimation error caused by concrete variables. The main difference between the two models is that the fixed effect model treats the unobserved differences between individuals as fixed parameters. In contrast, the random effect model treats the missing variables as random variables with a special probability distribution and assumes they are unrelated to the observed variables. This assumption of the stochastic model is generally challenging because the missing variables are usually associated with other explanatory variables in the model. The Hausman test testifies the null hypothesis that the random effects coefficients are identical to the fixed effects coefficients. The test produced *P* < 0.001 for both SRH/BMI, indicating that fixed effect models were appropriate.

To address the potential endogeneity, one of the best methods for addressing confounding in observational research are fixed effect models applied to longitudinal data containing repeated measures of both income and health (27). In this study, a longitudinal linear fixed-effects regression model was employed to estimate the association between changes in annual family income per capita and changes in SRH/BMI during three waves in older adults. The fixed effect model could effectively eliminate the influence of missing variables on dependent variables and the interference between independent and dependent variables. Meanwhile, the model also allows us to control time-variant factors that do not vary across individuals.

Specification of our model was as follows:

SRH_*it*_ = β_0_ + β_1_ Annual family income per capita_*it*_ + β_2_ Control A_*it*_ + β_3_ Control B_*i*_ + ε_*it*_
BMI_*it*_ = β_0_ + β_1_ Annual family per capita_*it*_ + β_2_ ControlA_*it*_ + β_3_Control B_*i*_ + ε_*it*_

SRH_*it*_ refers to SRH for individual *i* at time *t*, and BMI_*it*_ refers to BMI for individual *i* at time *t*. Similarly, Annual family income per capita_*it*_ denotes the explanatory variable, the annual family income per capita for individual *i* at time *t*. Control *A*_*it*_ represents the time-varying control variable, including age, marital status, and chronic disease. Control *B*_*i*_ represents the non-time-varying control variable, including gender and the number of years of education. ε_*it*_ is the error term.

## Results

### Basic Characteristics of the Respondents

Table 1 shows descriptive statistics separated by sample wave. Overall, the SRH increased significantly from 2010 to 2014, and the average value in 2018 was the same as that in 2014. For BMI, the rural elderly had a slight increase with age.

**Table 1:** Characteristics of the respondents.

**Variable**	**2010**		**2014**		**2018**	
	***n*** **(%)**	***M*** **(** ***SD*** **)**	***n*** **(%)**	***M*** **(** ***SD*** **)**	***n*** **(%)**	***M*** **(** ***SD*** **)**
SRH		2.16 (1.17)		3.54 (1.25)		3.54 (1.28)
BMI		21.89 (3.28)		21.93(3.97)		22.52 (4.05)
The annual family income per capita		5401.40 (7137.36)		6902.47 (7832.86)		6090.83 (8717.06)
Age		65.00 (4.35)		69.00 (4.35)		73.00 (4.35)
The number of years of education		3.22 (3.84)		3.22 (3.84)		3.22 (3.84)
Gender						
Female	279 (42.40)		279 (42.40)		279 (42.40)	
Male	379 (57.60)		379 (57.60)		379 (57.60)	
Marital status						
Married	561 (85.26)		527 (80.09)		503 (76.44)	
Divorce/Separate/Widowed	97 (14.74)		131 (19.91)		155 (23.56)	
Chronic disease						
Yes	144 (21.88)		191 (29.07)		192 (29.33)	
No	514 (78.12)		466 (70.93)		465 (70.67)	

The annual family income per capita we used was comparable with that in 2010. The revenue increased significantly from 2010 to 2014 and decreased slightly in 2018.

### Regression Results of the Effect of Annual Family Income per Capita on SRH and BMI

Model 1 (Table 2) summarized the modeling results of SRH changes in 2010, 2014, and 2018 and changes in equivalized annual family income per capita in 2010, 2014, and 2018, controlling for survey year, age, and marital status. The annual family income per capita was not significantly associated with SRH in model 1. Model 2 and 3 summarized the changes in SRH and equivalized per capita family income between illiterate and non-illiterate in 2010, 2014, and 2018, controlling for age, marital status, chronic diseases, and survey year. Interestingly, with the increase of annual family income per capita of the rural illiterate elderly, their SRH level improved significantly (β = 0.0770; 95% CI = 0.0065–0.1473), but for the non-illiterate elderly, there was no significant correlation between their SRH and annual family income per capita.

**Table 2:** Fixed effect modeling of changes in SRH and the annual family income per capita between 2010, 2014, and 2018.

	**Model 1: Whole sample**		**Model 2: illiteracy**		**Model 3: non-illiteracy**	
	**β**	**95% CI**	**β**	**95% CI**	**β**	**95% CI**
Ln (The annual family income per capita)	0.0338	−0.0182 0.0858	0.0770^**^	0.0065 0.1473	−0.0252	−0.1018 0.0514
Age	0.0132	−0.0957 0.1220	0.0210	−0.0919 0.1338	−0.1099	−1.0305 0.8107
Marital status	−0.2139	−0.5313 0.1035	−0.0189	−0.4267 0.3889	−0.4830	−0.9976 0.0223
Chronic disease	−0.3433^**^	−0.4782 −0.2082	−0.4844^***^	−0.6712 −0.2976	−0.1711*	−0.3643 0.0222
Intercept	1.5333	−6.4315 9.4982	0.5044	−7.7907 8.7996	11.1732	−55.8225 78.1689
*R*^2^: within	0.4110		0.3837		0.4614	

*Regression coefficients are unstandardized and unweighted;*

*** *P < 0.01;*

** *P < 0.05*.

Model 4 (Table 3) summarized the modeling results of BMI health changes in three waves and changes in equivalized annual family income per capita in three waves. The time-varying factors of survey year, age, and marital status were controlled. The annual family income per capita was significantly associated with BMI in model 4. Income increased corresponded to increases in BMI (β = 0.1614; 95% CI = 0.0325–0.2903).

**Table 3:** Fixed effect modeling of changes in BMI and the annual family income per capita between 2010, 2014, and 2018.

	**Model 4: Whole sample**		**Model 5: illiteracy**		**Model 6: non-illiteracy**	
	**β**	**95% CI**	**β**	**95% CI**	**β**	**95% CI**
Ln (The annual family income per capita)	0.1614^**^	0.0325 0.2903	0.2462^**^	0.05519 0.4372	0.0470	−0.1173 0.2113
Age	0.0357	−0.5257 0.5970	−0.0275	−0.6770 0.6220	1.0642	−0.8962 3.0246
Marital status	0.1061	−0.6935 0.9057	−0.0014	−1.1382 1.1354	0.3011	−0.7952 1.3973
Chronic disease	0.1246	−0.2116 0.4607	0.0252	−0.4806 0.5311	0.2436	−0.1769 0.6640
Intercept	18.4295	−22.5309 59.3897	22.1391	−25.3525 69.6306	−55.2547	−197.8846 87.3751
*R*^2^: within	0.0241			0.0237	0.0362	

*Regression coefficients are unstandardized and unweighted:*

** *P < 0.05*.

Model 5 and 6 (Table 3) summarized BMI changes and equivalent annual family income per capita between illiteracy and non-illiteracy in three waves. Compared with the overall rural elderly, the increase in BMI of the elderly in illiterate rural areas was more prominent (β = 0.2462; 95% CI = 0.05519–0.4372), and there was not significantly associated with BMI for non-illiteracy.

## Discussion

This study used a nationally representative dataset to investigate annual family income per capita on BMI and SRH among rural older adults in China. We found that, for SRH, with the increase of the annual family income per capita, the better results of SRH only existed in the illiterate elderly. However, for BMI, annual family income per capita had a positive correlation with the BMI of the rural elderly, that is, with the increase of income, the BMI of the rural elderly also increased. Meanwhile, in the illiterate elderly, with a rise in income, BMI was more prominent. Our application of dynamic panel models with fixed effects on the Chinese rural old adult population provided a unique chance to uncover the association between annual family income per capita and SRH and BMI in a society undergoing the rapid development of the rural economy.

In previous studies, the relationship between income and SRH was not consistent. Our research found that the increase of income among the rural elderly did not cause significant changes in SRH, and these changes only existed in the illiterate elderly after the stratification of education level. Gunasekara et al. analyzed 13 studies and five longitudinal surveys from four different countries. They came to a general conclusion: in most cases, there was a slight positive correlation between the increase of income and SRH, but these four countries were developed countries and did not consider developing countries (27). By analyzing the panel data of Canada, Veenstra et al. found a weak negative correlation between family income and self-health (18). However, the difference between our study results and the above is probably due to the different study objects. The population of our study is the elderly in rural areas. We can use resource substitution (28) to explain our results. When resources replace each other, the existence of one resource will reduce the harm caused by the lack of another resource. On the contrary, the less one resource, the more influential the other. Income and education are two kinds of resources. Using the resource substitution perspective, we can argue that more substantial health-protective effects of income among disadvantaged populations (rural illiterate elderly) because they have fewer alternative health-promoting resources, especially in rural areas. The increase of family income can increase their economic resources, better access to medical care, and more extensive social networks that promote healthier lifestyles that helps rural illiterate elderly to avoid poor health. Chronic diseases pose a great health threat to the rural elderly, reducing SRH, especially for the illiterate rural elderly. It can be explained that chronic diseases can lead to the decline of physical function of the elderly, affect their mental health, and reduce their quality of life (29).

The BMI of rural elderly increased significantly with the increase of income, but there is no such relationship among the non-illiteracy elderly. Our study differs from the conclusion of Deuchert et al., who found that obesity was a problem that focuses on the social and economic elites in developing countries, such as high-income groups (30). The previous explanation was that the relationship between income and BMI is related to nutrition change. The shift in nutrition refers to the change in eating habits with the development of the economy (31). Through the China Health and Nutrition Survey (CHNS) panel data, Ren found that rising income increased the adults' BMI and the propensity to be overweight. From the perspective of nutrition, there are five potential channels: nutritional intakes, dietary diversity, dietary knowledge, food preference, and dining out, among which dietary diversity plays the most significant role in explaining the income impact (32). It is also helpful to explain the relationship between family income and BMI in rural elders by nutritional change. Because for the rural elderly, higher family income means more food choices and dining out, increasing their extra energy intake. However, we were surprised to find that education seems to be alleviating this relationship, and the educated rural elderly do not increase BMI due to the increase of income. This result can explain the associations between levels of education and nutrition knowledge and concern with weight control (33).

Three limitations of this study must be noted. Firstly, because our BMI and SRH are self-reported—the prevalence of obesity would be overestimated when using self-reported height and weight (34), recall bias may exist in this study, affecting the accuracy of the estimation. Future research should consider other indicators, such as body fat percentage and waist-hip ratio, to better measure body fat and subjective health. At the same time, more perfect scales can be used to measure health status. Secondly, fixed effects analysis controls time-invariant confounding factors at the individual level. There may be unmeasurable or unmeasurable time-varying variables in this study. Third, our sample is restricted to better observe the changing process of the health impact of family income among the rural elderly in China. A threat to representativeness pertains to the high attrition rate experienced by the CFPS from wave one to wave three, although we control for marital status, gender, and age in our models. The existence of missing independent and dependent variables of the CFPS compromises the generalizability of our results, given that rural older are not well-represented by our study. Overall, our study has selection bias.

## Conclusion

Few studies measured the association between annual family income per capita between BMI and SRH in rural elderly using fixed effects analysis that rules out the potential endogeneity to the best of our knowledge. At the same time, this study considers the gap between illiteracy and non-illiteracy to adapt to the high illiteracy rate in rural areas. In this study, the illiteracy rate is as high as 55.47%.

Self-rated health increased with higher the logarithmized family income per capita among the rural illiterate elderly. Moreover, BMI increased with higher the logarithmized family income per capita among the rural elderly, especially the illiterate elderly.

In rural economic development, the impact of family income on the rural elderly is undeniable. It is worth noting that the health status of the rural illiterate elderly needs particular attention, because first of all, they have lower education levels and lack health knowledge. Second, most of them have lower insurance levels and lower ability to resist disease risk. At the same time, the average BMI of the rural non-illiterate elderly was 22.56 ± 3.67, while the average BMI of the rural illiterate elderly was 21. 75 ± 3.85. The family income of the illiterate elderly in rural areas also needs to be paid attention to because the increase of average family income can improve their BMI, which is likely to be reasonable. Theoretically and methodologically, we need more research to understand the relationship between socioeconomic status and the health of the elderly and its internal mechanism. It also reminds us to consider the educational differences in rural areas when investigating causal relationships further, which may help develop more targeted strategies in a developing country.

## Data Availability Statement

The original contributions presented in the study are included in the article/supplementary material, further inquiries can be directed to the corresponding author.

## Author Contributions

YX: methodology, software, and writing—original draft. XR: writing—review and editing. All authors have read and agreed to the published version of the manuscript.

## Conflict of Interest

The authors declare that the research was conducted in the absence of any commercial or financial relationships that could be construed as a potential conflict of interest.

## Publisher's Note

All claims expressed in this article are solely those of the authors and do not necessarily represent those of their affiliated organizations, or those of the publisher, the editors and the reviewers. Any product that may be evaluated in this article, or claim that may be made by its manufacturer, is not guaranteed or endorsed by the publisher.
